# Human food sources shape routine movements across elevational gradients in an alpine bird

**DOI:** 10.1186/s40462-026-00694-2

**Published:** 2026-08-26

**Authors:** Kristina B. Beck, Matthias-Claudio Loretto, Thomas Müller

**Affiliations:** 1 Senckenberg Biodiversity and Climate Research Centre, Frankfurt am Main, Germany; 2 Research Institute of Wildlife Ecology, Department of Interdisciplinary Life Sciences, University of Veterinary Medicine, Vienna, Austria; 3 Department of Biological Sciences, Goethe University Frankfurt, Frankfurt am Main, Germany

**Keywords:** Human food sources, Alpine chough, *Pyrrhocorax graculus*, Altitudinal movement, Alpine ecosystem, Site fidelity, Movement routine, GPS tracking

## Abstract

**Background:**

Human activities are reshaping animal movement patterns worldwide, with human food sources emerging as a significant driver. While such subsidies are known to reduce space use and alter migration behaviour, their role in shaping routine movement patterns—such as sequential visits to specific sites—remains poorly understood.

**Methods:**

Here, we investigated the impact of human food sources on the movement patterns of Alpine choughs (*Pyrrhocorax graculus*), a high-elevation corvid that readily exploits human food. In winter, these birds engage in altitudinal movements, descending from alpine areas to lower elevations in search of food. Thereby, they encounter diverse human food sources ranging from mountain restaurants at high altitudes to urban garbage dumps and bird feeders in the valleys. We analysed the movements of 40 GPS-tagged individuals and combined tracking data with behavioural observations to (1) quantify the occurrence and spatiotemporal patterns of altitudinal movements (2), assess the extent to which human food sources shape space use, and (3) identify potential recursive movement patterns.

**Results:**

Alpine choughs performed daily altitudinal movements during winter, spanning up to 3000 m in elevation per round trip. Birds typically descended in the early morning and returned upslope around midday. Their movements consistently overlapped with human-modified environments providing human food sources, including towns, villages, and mountain restaurants. Notably, individuals repeatedly visited four main foraging locations associated with human food, showing strong route fidelity by returning to the same food-provisioning sites in a consistent sequence and at predictable times of day.

**Conclusions:**

Our findings highlight how human food sources can shape population-level movement patterns and foraging routines, with potential ecological consequences for species increasingly reliant on human-modified habitats. Such dependence may limit behavioural flexibility and create ecological traps, where individuals repeatedly exploit risky or suboptimal resources. By moving daily between alpine and urban areas, Alpine choughs may also transport seeds, nutrients, and pathogens across ecosystems, generating cascading effects in both mountain and lowland habitats.

**Supplementary Information:**

The online version contains supplementary material available at 10.1186/s40462-026-00694-2.

## Background

Human activities are profoundly altering animal movement patterns across the globe, with cascading effects on animal populations and whole ecosystems [1–3]. From habitat fragmentation and infrastructure development, which create barriers and alter movement routes [4, 5], to pollution hindering animal navigation and movement decisions [6, 7], the footprint of human influence is increasingly evident in the spatial ecology of wildlife. Thus, developing a deeper understanding of how anthropogenic activities shape animal movement is crucial for predicting ecological consequences and maintaining population viability.

A fundamental facet of anthropogenic influence is the provision of supplemental food sources. Human food sources range from intentional feeding of wildlife, to garbage, food scraps, fishery discards, and agricultural commodities, which can dramatically alter animal movement [8–10]. Various species show strong reductions in their overall space use when supplemental food sources are present [11–13], and several migratory birds have been observed to shorten or abandon their migrations in response to readily available human food sources [2]. Such effects have been documented across ecosystems, including: urban environments, where animals make use of available refuse and supplemental feeding [14, 15]; agricultural landscapes, where ungulates and birds adapt their movements to exploit crop residues and supplemental feed provided for livestock [16, 17]; and coastal ecosystems, where marine birds and mammals change their foraging habits and distributions due to fishery discards [11, 18].

A key driver of these movement changes is likely the high spatiotemporal predictability of human food sources [9, 19]. Predictable resources allow animals to rely on spatial memory, leading to the repeated use of specific sites and the emergence of recursive movement patterns [19–21]. This in turn can give rise to routine movements—sequential and repeated visits to multiple, known locations [19, 22]. Human food sources may thus not only result in a mere reduction of space use but also potential changes in how animals organize their movement over time. Such human-induced movement routines may have cascading effects on species’ energy budgets, intra- and interspecific interactions, and even evolutionary consequences. Yet, while the effects of human food sources on general space use are well documented in some species, we know little about whether—and how—the predictability of these resources shapes the development of routine movement patterns in real-world systems.

In this study, we investigate the impact of human food sources on the movement patterns of the Alpine chough (*Pyrrhocorax graculus*), a corvid inhabiting high-elevation habitats. While their natural diet consists primarily of berries and invertebrates, Alpine choughs readily exploit human food sources, including garbage, food scraps, and direct handouts from humans [23]. Particularly during winter, these birds engage in altitudinal movements descending to lower elevations in search of food, encountering a range of human food sources—from mountain restaurants at higher altitudes to garbage dumps and bird feeders in urban areas in the valleys [23]. Previous observational studies suggest that Alpine choughs may undertake regular, daily foraging trips along this altitudinal gradient, often returning to the same sites [23, 24]. However, detailed individual-level tracking data are lacking, and the spatiotemporal patterns of these altitudinal movements remain poorly understood.

By combining behavioural observations with fine scale GPS tracking, we aimed to investigate: (1) the occurrence and the spatiotemporal patterns of altitudinal movements; (2) the extent to which human food sources influence the birds’ space use; and (3) identify potential recursive movement patterns. We hypothesize that human food sources, across elevational gradients, will significantly influence the space use of Alpine choughs during altitudinal movements by attracting them to predictable locations with human food availability, thereby promoting the development of routine movement patterns.

## Methods

### Study species and site

The study was conducted from 2023 to 2025 on a population of Alpine choughs in the Northern Alps of Austria and Germany (coordinates: 47.574598, 13.042455). Alpine choughs are a medium-sized corvid inhabiting high-altitudinal environments across Europe, Morocco, and Asia [25]. They are highly social birds, occurring in groups of varying sizes throughout the year [26]. During the breeding season (May-July), Alpine choughs typically nest in cliff faces and rock crevices at high elevations. They are socially monogamous, with pairs raising clutches of up to five chicks [27].

The study site is characterized by rugged terrain, including steep cliffs, rocky outcrops, and alpine meadows, spanning a range of elevation zones, each characterized by distinct climatic conditions and vegetation structures. Following the classification of König et al. [28], we defined five elevation zones: submontane (600–800 m above sea level; hereafter m a.s.l.), montane (800–1200 m a.s.l.), high montane (1200–1500 m a.s.l.), subalpine (1500–1800 m a.s.l.), and alpine (1800–2500 m a.s.l.). The tree line in the region is generally found around 1500 m a.s.l [29]. Within the study area, various human food sources are present across the elevational gradient. These include urban and rural settlements at lower elevations (e.g., villages, towns below 1200 m a.s.l.), and mountain huts and restaurants located at higher elevations within the alpine and subalpine zones.

### Capture and data collection

**Fig. 1:**
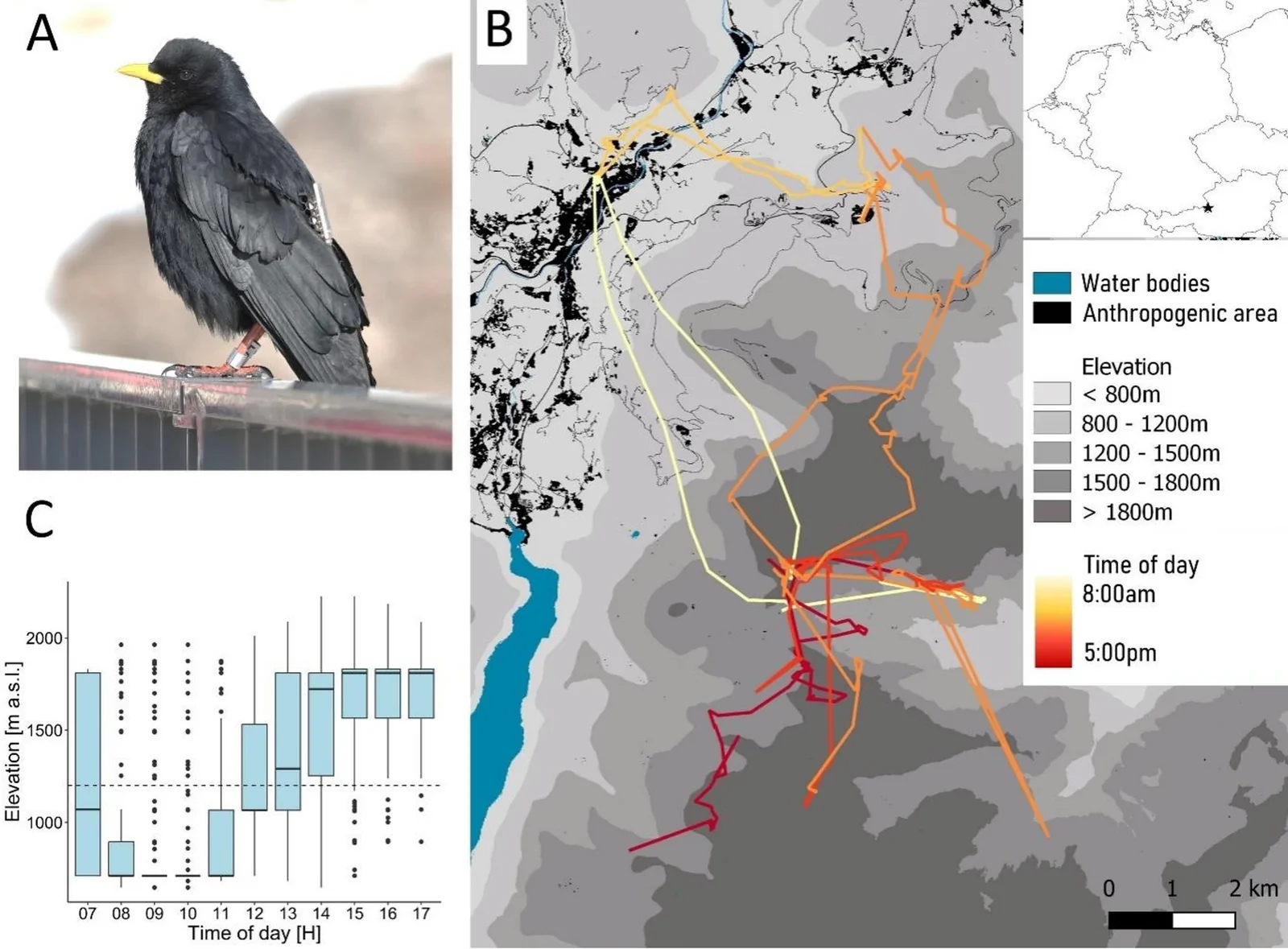
Daily altitudinal movements of GPS-tracked Alpine choughs. **A**: GPS-tagged Alpine chough. **B**: Examples of high-resolution trajectories of daily altitudinal movements by two individuals (randomly selected). Colour represents time of day, ranging from 8 am (bright yellow) to 5 pm (dark red). Water bodies are shown in blue, anthropogenic structures such as buildings and roads are shown in black. The different shades of grey illustrate changes in elevation (meters above sea level; m a.s.l.). The map in the right corner shows Germany, with the asterisk indicating the study site. **C**: Daily variation in elevation during altitudinal movements averaged across the whole population

From May 2023 – March 2024, we equipped 54 Alpine choughs with GPS-transmitters (Fig. 1A; GPS-Bluetooth-Solar NANO tracker, Druid Technology). Birds were caught using woosh nets and cage traps baited with food, and we marked them individually with a unique metal leg ring and plastic colour ring with a unique alphanumerical code. We measured tarsus length and body weight, and took a small blood sample for sexing (19 females, 35 males). Birds were fitted with a GPS-transmitter using leg-loop harnesses built from Teflon ribbon and aluminium crimps. The combined weight of the transmitter and harness was approximately 5 g, representing less than 5% of the average body mass of Alpine choughs (230 g). The GPS-transmitters are solar-powered and collected GPS-positions in different intervals depending on voltage level (1 GPS/min up to 1 GPS/3 h, with occasional longer gaps of several days). Daily GPS recording began earliest at 7 am to latest 5 pm and data collection was switched off during the night to save battery.

### Analysis

#### Occurrence and spatiotemporal patterns of altitudinal movements

For all our analyses, we focused on the winter period (November to April), when Alpine choughs are known to undertake daily altitudinal movements. To quantify the extent of these movements, as well as their spatiotemporal patterns, we combined GPS tracking with behavioural observations.

For the tracking data, we analysed the movements of 40 birds between November 2023 and April 2024 (14 individuals ceased transmitting spatial locations prior to the winter period and were therefore excluded from the analysis). We defined altitudinal movements as any instance in which an individual moved into the montane zone, below 1200 m a.s.l. This threshold reflects a marked shift in both elevation and habitat type (i.e., throughout the year, choughs typically occurred at elevations above 1655 m a.s.l. (Additional file 1: Fig. S1), indicating a primary association with subalpine and alpine zones). Following that, we estimated both the number of days during which altitudinal movements occurred and the proportion of individuals exhibiting them. Further, we inferred the extent of altitudinal movements at the individual level as the proportion of days during which a bird was recorded below 1200 m a.s.l. To ensure reliable inference of daily altitudinal movements, we restricted the analysis to days with at least three spatial fixes, including at least one fix in the morning to reduce the likelihood of missing altitudinal descents. In addition, we included only individuals with a minimum of 10 altitudinal trajectories (approximately the 2.5% quantile of all individuals), ensuring that estimates were based on sufficiently long and representative tracking histories of individuals.

To infer the spatiotemporal distribution of birds during altitudinal movements, we selected, for each individual, only those daily trajectories that included at least one GPS location below 1200 m a.s.l., indicating a descent to lower elevations. To avoid bias arising from differences in GPS sampling frequency, we further subsampled all tracking data to an hourly interval and retained only individuals with at least 55 relocations (25% quantile) across the winter period, resulting in a final dataset of 31 individuals (relocations: min = 57, mean = 304, max = 592). We then estimated the space use of each individual, by calculating the 95% and the 50% utilisation distribution (UD) using the function “getVolumeUD” based on dynamic Brownian Bridge Movement Models (dBBMM) from the “move” R package [30]. A utilisation distribution describes the probability of where an individual occurs in space at any randomly chosen moment. To estimate the dBBMMs, we used the following parameters: margin of 11, window size of 31, an estimated location error of 10 m, raster cell size of 100 m, “timestep” as 1 min, coordinate reference system: EPSG: 32,633, and for calculating the variance, we excluded time lags that exceeded 3 h. No additional boundary constraints (e.g., elevation masks or predefined study-area polygons) were applied; thus, UDs were estimated directly from the observed movement data.

To complement the GPS data—given that only about 30% of the local population was tagged—we also conducted behavioural observations in the valley at the town of Berchtesgaden (approx. 650 m a.s.l.) where Alpine choughs regularly occur during winter. Observations were carried out daily from February to April in both 2024 and 2025 by two observers. One observer was stationed at an elevated vantage point overlooking much of the town to track flock movements and presence, while a second observer followed the flock by bicycle to obtain more detailed information on behaviour and food sources. During each observation day, we recorded whether Alpine choughs were present in the town, flock size, arrival and departure times, their spatial locations, behavioural activities (e.g., feeding and resting), and associated food sources. Data on the presence and flock sizes of Alpine choughs were used to infer the extent of altitudinal movements. Spatial location data were used to quantify fine-scale space use within the town of Berchtesgaden by calculating 95% and 50% minimum convex polygons (MCP) using the “adehabitatHR” package [31]. Together, these behavioural observations provided detailed information on low-elevation space use, complementing the broader-scale movement patterns derived from GPS tracking data. Comparable behavioural observations were not conducted at other sites.

#### Human impact on the birds’ space use during altitudinal movements

We examined the extent to which GPS-tracked birds occur in human-modified versus natural habitats during altitudinal movements using a logistic regression (“lme4” R package [32]), comparing habitat characteristics of observed and random spatial points. To draw random points, we first calculated a 100% MCP from all observed spatial locations to cover the birds’ activity range. We then generated random points (ten times as many points as observed) within the estimated activity range using the “amt” R package [33]. For each “observed” and “random” point, we then extracted the underlying habitat type. Habitat categories were derived from Marsoner et al. [34] which catalogues 65 habitat classes with a spatial resolution of 5 m. For our purpose, we grouped all habitat classes into two categories: “human (e.g., settlements, roads)” and “natural (e.g., forests, grasslands)”. We then fitted the “occurrence” (1 = observed, 0 = random) as the dependent variable and habitat category (human/natural) as the explanatory variable. To facilitate interpretation, model predictions were transformed from the logit scale to the probability scale, yielding predicted probabilities of habitat use for each habitat category.

Finally, to complement the habitat selection analysis and identify the resources underlying birds’ use of low-elevation habitats, we conducted behavioural observations in the town of Berchtesgaden. Food items consumed and their corresponding foraging locations were classified as either human-derived (e.g., bird feeders, food provided by people, or food waste) or natural (e.g., foraging in meadows), allowing us to quantify the relative contribution of human and natural food resources.

#### Recursive movement patterns

We aimed to examine whether Alpine choughs repeatedly visited sites associated with human food resources and whether these sites were visited in a consistent sequence, indicative of routinized movements. To address this, we (1) identified and classified recurrent revisitation sites as either human-food associated or not, and (2) quantified the sequential order and timing of visits to these sites.

##### (1) Identification and classification of revisitation sites

We identified recurrent sites by applying a 500 m radius around each GPS location and defining a “revisit” as any subsequent location falling within this radius, using the R package “recurse” [35]. The 500 m threshold was chosen to approximate the spatial scale of typical foraging areas in the study system, including urban habitats and point-like anthropogenic food sources such as mountain restaurants. This choice was further supported by independent behavioural observations in the town of Berchtesgaden, where the 95% MCP of observed foraging locations spanned approximately 500 m in both dimensions (see Results and Additional file 1: Fig. S2). For each GPS location, we calculated the total number of revisits and retained locations accounting for at least 50% of all revisits. Analyses were restricted to trajectories classified as altitudinal movements, and all GPS data were subsampled to an hourly interval to ensure consistent sampling effort among individuals. 

For each individual, frequently revisited locations were subsequently grouped into discrete revisitation sites using density-based spatial clustering (R package “dbscan” [36]) with a neighbouring radius of 500 m (eps). To characterize the habitat composition of each revisitation site, we calculated a 100% MCP encompassing all locations within each cluster and quantified the proportional overlap of the resulting polygon with human and natural habitat classes (see habitat classification above). Finally, revisitation sites were classified as either human-food associated or non-human-food associated based on habitat composition and visual inspection of the surrounding spatial context (e.g., the presence of mountain restaurants).

##### (2) Temporal structure of revisitation patterns

To identify potential routine movement patterns among revisitation sites, we analysed the temporal structure of daily visitation sequences. We retained only daily trajectories containing at least 10 GPS locations and included only individuals with at least 10 such days (*N* = 23 individuals), ensuring sufficient data to characterize within-day movement sequences. We then quantified (i) the probability that an individual visited any identified site on a given day, (ii) site-specific visitation probabilities, (iii) the consistency of sequential visitation order among sites, and (iv) the timing of visits within the day.

## Results

### Occurrence and spatiotemporal patterns of altitudinal movements

Altitudinal movements were recorded on 162 out of 176 days (92%). This pattern was corroborated by behavioural observations in the town Berchtesgaden where Alpine choughs were observed foraging on 122 out of 136 observation days (90%). The proportion of GPS-tagged birds recorded below 1200 m a.s.l. (min = 0%, mean = 59% max = 100%), as well as the maximum number of choughs counted during observations (min = 0, mean = 105, max = 224), varied. At the individual level, all birds undertook altitudinal movements on an average of 56% of observation days (min = 13%, max = 90%).

Birds typically arrived at lower elevations early in the morning and returned to higher elevations around noon or in the early afternoon, covering elevation changes of more than 3000 m in a single round trip (Fig. 1B, C). Most descents occurred before the GPS units became active or when energy levels were insufficient for data transmission. However, based on behavioural observations in the town Berchtesgaden, birds arrived until 8/9 am or were already present before observations started (i.e., before 7:30 am).

The birds’ local activity range (95% UD) during altitudinal movements averaged 84.74 km² (range: 60.36–141.81 km²), with a mean core area (50% UD) of 18.73 km² (range: 2.70–29.38 km²). Activity ranges spanned various elevations, with the majority occurring at low (< 1200 m) and high elevations (> 1500 m; Additional file 1: Fig. S3). Within the town of Berchtesgaden, Alpine choughs covered an area of approximately 0.17 km² (95% MCP) and 0.05 km² (50% MCP), as inferred from behavioural observations (for each year separately: 95% MCP: 0.16 km² for 2024 and 0.15 km² for 2025; 50% MCP: 0.05 km² for 2024 and 0.04 km² for 2025; Additional file 1: Fig. S2).

#### Human impact on the birds’ space use during altitudinal movements

Alpine choughs were more likely to be present in human-modified environments than in natural habitats (Estimate ± standard deviation: -2.90 ± 0.03, z value: -104.86, *P* < 0.001). The probability of encountering an Alpine chough in human-dominated spaces was 53%, compared to 6% in natural habitats.

Behavioural observations in the town Berchtesgaden yielded a total of 1079 feeding events, including both clearly and ambiguously identifiable food sources. In 42% of cases, birds were observed feeding directly on human food, including bird feeders (32.44%), direct feeding by humans (5.38%), and other human-derived resources such as bread or waste on streets (4.17%). A further 35.77% of feeding events occurred in urban green spaces such as gardens and meadows, where birds likely consumed natural food items including fruits, seeds, and arthropods. In 12.88% of cases, birds foraged on building surfaces (walls and roofs), where the exact food source could not be identified but may have included arthropods or mineral substrates such as chalk. An additional 8.43% of observations involved foraging on streets with unidentified food items, and in 0.93% of cases birds were observed consuming conspecific or their own faeces [37].

#### Recursive movement patterns

**Fig. 2:**
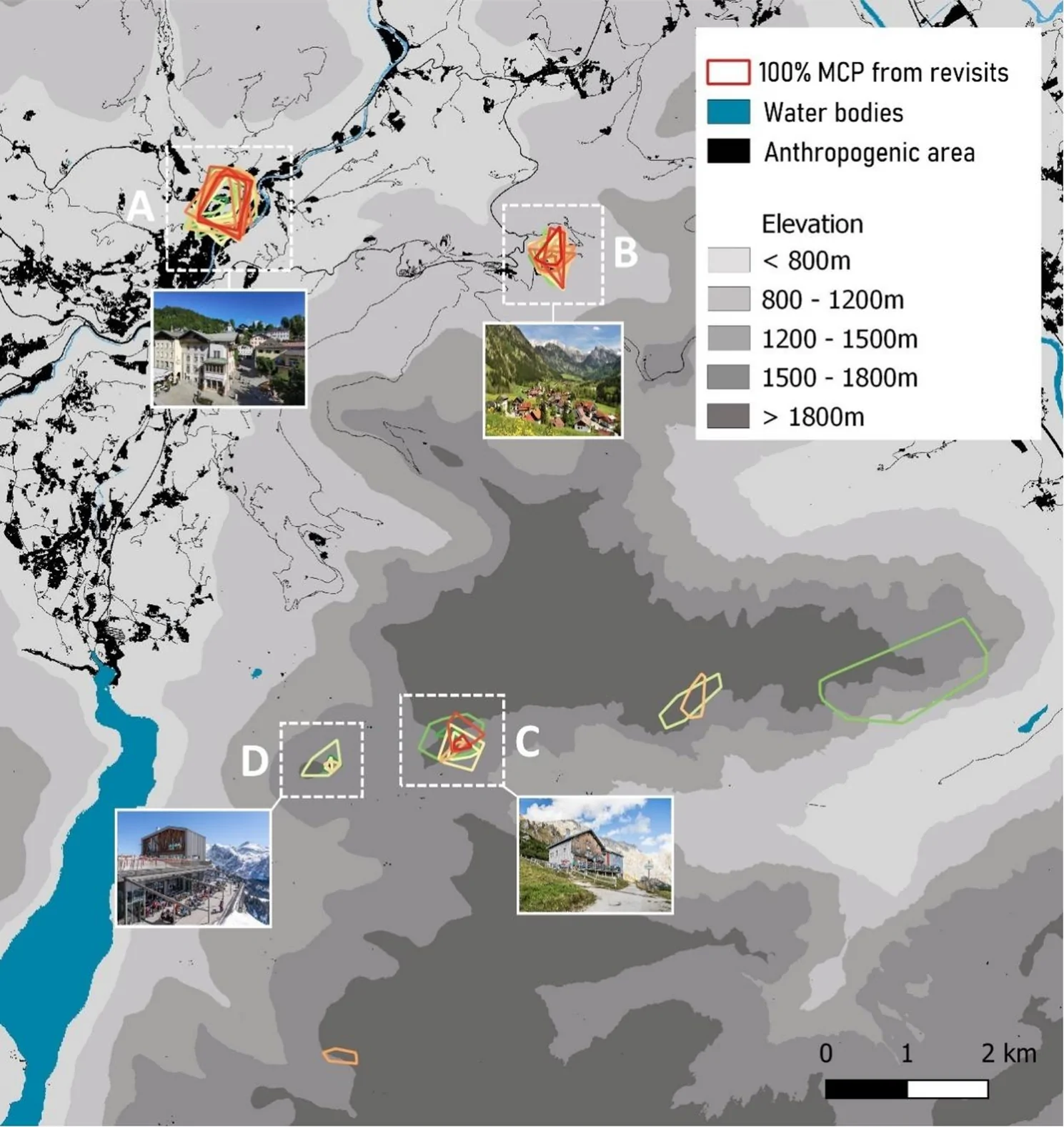
Map showing frequently revisited sites. Polygons show the 100% minimum convex polygons (MCPs) inferred from the 50% top revisited sites for each individual (different colours for each individual). For visualization purposes two revisitation sites of one individual are not shown. White, dashed rectangles illustrate four locations that were frequently visited by more than one GPS-tracked bird. Pictures show the anthropogenic structures at each site, and potential associated human food source (two urban spaces at low elevation, and two mountain restaurants at high elevation). Water bodies are shown in blue, anthropogenic structures such as buildings and roads are shown in black. The different shades of grey illustrate changes in elevation

We identified nine frequently revisited sites (with more than 50% of all revisits, see methods; Additional file 1: Fig. S4) of which five overlapped with anthropogenic structures related to human food (i.e., towns/villages, mountain huts/restaurants; Fig. 2). All identified “natural” sites were located at high altitudes while all frequently revisited sites below 1200 m a.s.l. were associated with human food (i.e., towns/villages). Four of the nine clusters were consistently visited by at least four GPS-tracked birds (Fig. 2) and were all associated with human food, including two urban lowland sites (site A: 30 individuals; B: 17 individuals) and two high-elevation sites (site C: 7 individuals; D: 4 individuals; Fig. 2).

From 1949 altitudinal movement trajectories, we selected 527 high resolution trajectories ( > = 10 GPS locations, >= 10 days, *N* = 23 individuals) to identify the number and sequential order in which the four main identified human food sources (A-D) were visited. Alpine choughs visited on average 2 of the 4 identified food sources on the same day (min = 0, max = 4), travelling an estimated minimum of approximately 20 km when visiting all four identified human food locations, based on the cumulative straight-line distances from the mountain top to the first feeding site (site A) and between successive feeding sites. Across all individuals, the probability to visit any of the four sites on a given day was 94% (95% Confidence Interval (hereafter 95% CI) = 88–100%; min = 38%, max = 100%). On average, individuals visited on 86% of observation days site A (95% CI:78–93%; min = 30%, max = 100%), in 65% of cases site B (95% CI:59–72%; min = 21%, max = 85%), in 29% site C (95% CI:25–33%; min = 8%, max = 47%), and in 21% site D (95% CI:10–32%; min = 0%, max = 92%).

**Fig. 3:**
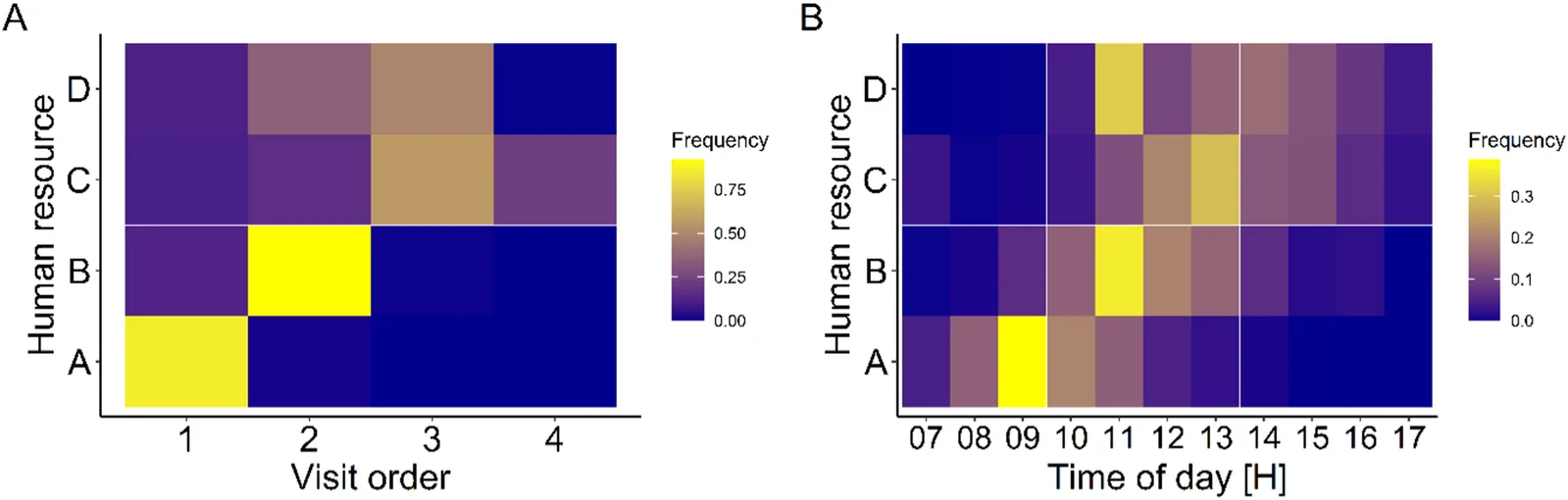
Order and timing of visits to human food sources.** A**: Visit order to the identified four main human food sources A-D. **B**: Time of day (in hours) during which each of the four human food sources A-D was visited. Colour represents the proportional frequency of each visiting order/time of day for each of the four human food sources averaged across all individuals. The four human food sources are ordered by elevation (increase from A to D)

Regarding the visiting order across all individuals, site A was visited first in 86% of cases, and site B second in 92% of cases. In 57% and 49% of cases, site C and D were visited third (Fig. 3A, Additional file 1: Supplementary table S1, Additional file 1: Fig. S5 showing examples of the visitation order across food sources for four individuals separately). The order in which the four sites were visited differed significantly from uniform (X-squared = 1457.7, df = 9, p-value < 0.001), and were also not visited randomly across the day (X-squared = 1419.7, df = 30, p-value < 0.001). Site use over the day largely followed the visiting order, with peak use at site A around 9 am (39%), site B around 11 am (36%), site C around 1 pm (29%), and site D around 11 am (32%).

## Discussion

Here, we reveal detailed patterns of altitudinal movements in the Alpine chough, using GPS tracking. Our findings confirm previous hypotheses that choughs engage in regular daily altitudinal movements—descending to lower elevations in the early morning and returning to high-altitude areas by noon or early afternoon. Importantly, these movements were not random but highly structured and spatiotemporally predictable, with birds consistently selecting human-modified spaces and sequentially revisiting a limited number of specific sites.

Throughout winter, Alpine choughs performed daily altitudinal movements, typically foraging in urban areas during the morning before returning to high elevations by midday (Fig. 1). Alpine ecosystems pose significant challenges to animal survival, with harsh climates, limited resource availability, and seasonal snow cover constraining foraging and movement. Many alpine species thus perform altitudinal movements to cope with food scarcity, seeking milder conditions and greater food availability at lower elevations [38]. We hypothesize that environmental factors—particularly snow cover and food scarcity—drive the winter altitudinal movements observed in Alpine choughs, as previously documented for a population in the Italian Alps [24]. In winter, Alpine choughs are reported to feed primarily on berries such as Common Barberry (*Berberis vulgaris*) and Juniper (*Juniperus* spp.) [39]. However, these resources are largely absent in our study area [40], and deep snow cover, often several meters deep, likely exacerbates food limitation at higher elevations. In contrast, lower elevations offer more accessible fruiting vegetation, often remain snow-free, and provide predictable human food sources. Supporting this, we observed that, at one low-elevation site (site “A”), birds foraged on a range of human-derived food items (e.g., waste and bird feeders) and used snow-free green spaces that may provide access to fruits, seeds, and invertebrates.

Altitudinal movements are typically seasonal and cyclic (i.e., altitudinal migration [38]) where animals descend to lower elevations for the duration of winter (e.g. roe deer (*Capreolus capreolus*) [41] and mountain goats (*Oreamnos americanus*) [42]). Daily altitudinal movements, such as observed in Alpine choughs (Fig. 1), are rather rare. Comparable behaviours have been reported in ring ouzels (*Turdus torquatus*), which forage at lower elevations during the day while roosting at higher altitudes at night [43], and in Guizhou snub-nosed monkeys (*Rhinopithecus brelichi*), which reverse this pattern by ascending daily to forage and descending in the afternoon [44]. However, the consistent use of anthropogenic environments during such daily altitudinal movements, as seen in Alpine choughs, appears rare.

Food availability at high elevations may be particularly limited during early morning hours due to cold temperatures and the absence of human recreational activity (and thus human-derived food), which increases toward midday. Alpine choughs may therefore descend to lower elevations in the early morning to exploit more reliable food sources (e.g., bird feeders) that are available all day. It remains unclear why choughs return to higher elevations the same day rather than remaining in the lowlands for several days or across winter, as many other species do [38]. We speculate that daily ascents are associated with increased predation risk in the lowlands—where predators such as eagle owls (*Bubo bubo*), sparrowhawks (*Accipiter nisus*), and stone martens (*Martes foina*) are more prevalent—or by the lack of suitable roosting sites. Alpine choughs are known to roost in caves [23], which are largely absent in lowland areas and may offer thermal and protective advantages. In other species, similar trade-offs have been documented. For example, pregnant bighorn sheep (*Ovis canadensis*) migrate upslope earlier in spring than non-pregnant individuals to give birth in terrain that offers better protection from predators, despite the higher forage quality at lower elevations [45].

During altitudinal movements, Alpine choughs exhibited a strong preference for human-modified areas. At lower elevations, individuals were almost exclusively observed in urban environments, while at higher altitudes, they frequently occurred near mountain huts (Fig. 2, Additional file 1: Fig. S2, S3). Notably, birds displayed a high spatiotemporal fidelity to a limited set of human-associated sites (Fig. 2, Additional file 1: Fig. S4), mirroring routine-based foraging patterns. We recorded consistent visits by the majority of the population to four human sites—two in urban and two in alpine area—following a regular spatiotemporal sequence that coincided with altitudinal shifts (Figs. 2 and 3). Our findings thus go beyond opportunistic foraging, suggesting that predictable human food sources can shape highly structured daily routine movements in wildlife.

Our GPS tags were programmed not to record locations between 5 pm and 7 am and generally provided fixes at hourly intervals. In addition, no behavioural observations were conducted during this time at locations B, C, and D. Consequently, we cannot determine the food consumed at these sites, the exact duration of visits, or completely exclude the possibility that birds visited these locations while the GPS tags were inactive. However, this scenario appears unlikely given the short winter photoperiod (approximately 7:30 am–4:30 pm), which leaves little time for additional visits outside the movement patterns captured by the GPS data. Furthermore, the few complete downslope trajectories available suggest that birds generally moved directly to lower elevations rather than making prolonged stops at intermediate sites (Fig. 1B).

The recorded routine movement behaviour likely increases foraging efficiency by minimizing search time and maximizing energy intake, and can be characteristics of memory-based foraging strategies and spatial routines observed in other species [21, 46]. For example, Roe deer (*Capreolus capreolus*) rely heavily on recent experience to guide their foraging decisions, allowing them to adapt to changing resource availability [47]. Furthermore, abiotic factors can influence site/route fidelity; for instance, different species of birds of prey choose oceanic migratory corridors that offer optimal wind support and thermal uplift to minimize energy expenditure [48]. Finally, in socially living species like Alpine choughs, social learning may further reinforce fidelity to profitable routes and foraging locations across individuals and generations. In Whooping cranes (*Grus americana*), for example, juveniles flying with experienced adults adopt more efficient migratory paths, illustrating the importance of social transmission in shaping movement behaviours [49].

Future studies incorporating fine-scale modelling of abiotic environmental conditions, as well as experimental translocations or social learning experiments, could help elucidate the underlying mechanisms driving the observed population-level route fidelity in Alpine choughs. Further, it remains unclear to what extent the observed highly structured, daily movements are specific to our study population or shared across the species’ range. Comparative studies across different populations—exposed to varying levels of human impact, predator density, and (natural) food availability—are needed to assess the plasticity and underlying drivers of the species’ movement strategies.

While population-level routine behaviour likely provides several benefits, it also carries potential downsides. High route fidelity, especially when associated with human food, may reduce behavioural flexibility, making individuals less responsive to changes in natural food availability or environmental conditions [19]. Such specialization can lead to a form of ecological trap [50], where animals repeatedly exploit predictable but suboptimal or risky resources. A high dependence on human food may leave individuals vulnerable to abrupt changes in resource availability driven by shifts in waste-management practices and tourism intensity with cascading effects on movement behaviour and population dynamics [51]. For example, improvements in waste management at mountain huts, and the resulting reduction in predictable food subsidies, may decrease route fidelity and increase reliance on natural foraging areas (where available), potentially leading to more extensive or variable space use [51]. In addition, reliance on human-derived food can negatively impact individual health through reduced nutritional quality, increased exposure to contaminants or pathogens, altered gut microbiota, and potentially predation risk [9, 52–54]. Future research should therefore examine how dependence on human food shapes individual health, movement behaviour, and population dynamics, particularly in response to changes in the availability of human food sources.

Daily altitudinal movements, such as performed by Alpine choughs, can also amplify the ecological consequences of cross-ecosystem movements. By regularly transporting materials such as seeds, pathogens, or nutrients between urban and alpine zones, choughs may influence ecological processes in both habitats [55–57]. GPS-tracking of yellow-legged gulls (*Larus michahellis*) revealed that birds potentially dispersed seeds (including alien species) both within and outside of urban environment [58]. Similarly, Alpine choughs may contribute to the spread of non-native species encountering at peoples’ gardens from urban into alpine ecosystems. Such cross-boundary movements may alter nutrient dynamics and community composition in sensitive mountain habitats, particularly in the light of climate change [56, 57]. Understanding the ecological consequences of such movements—both beneficial and detrimental—will be essential for the future protection of alpine ecosystems.

## Conclusions

Our study demonstrates that Alpine choughs perform highly structured daily altitudinal movements that are closely linked to predictable human food sources. Rather than moving opportunistically, individuals repeatedly followed consistent routes and revisited the same limited number of human-modified sites at similar times of day across an urban–alpine gradient. These findings show that reliable human food sources can shape not only space use but also the temporal organisation of movement behaviour, generating stable, population-level routines, as well as reshape ecological connectivity. As environmental change and human influence continue to transform natural ecosystems, understanding the role of human food sources in shaping wildlife movements will be essential for predicting how species adapt to—or become increasingly dependent on—anthropogenic environments.

## Supplementary Information

Below is the link to the electronic supplementary material.

Supplementary Material 1: Additional file 1: Supplementary figures and tables accompanying the results presented in the main text.

## Data Availability

All data and R code to reproduce the results presented in this study are available at the Open Science Framework: 10.17605/OSF.IO/39MNB.

## Abbreviations

m a.s.l.: Meters above sea level
