# Supplementary material for: Human food sources shape routine movements across elevational gradients in an alpine bird
Source: Mov Ecol. 2026 Aug 26;14:60. doi: 10.1186/s40462-026-00694-2 (PMC13520249; doi:10.1186/s40462-026-00694-2)
Supplement: Supplementary file 1 [file 40462_2026_694_MOESM1_ESM.docx]

**Supplementary material for:** Human food sources shape routine movements across elevational gradients in an alpine bird

Kristina B. Beck^1*^, Matthias-Claudio Loretto^2^, Thomas Müller^1,3^

^1^ Senckenberg Biodiversity and Climate Research Centre, Frankfurt am Main, Germany

^2^ Research Institute of Wildlife Ecology, Department of Interdisciplinary Life Sciences, University of Veterinary Medicine, Vienna, Austria

^3^ Department of Biological Sciences, Goethe University Frankfurt, Frankfurt am Main, Germany

* corresponding author: kbbeck.mail@gmail.com

**Figure S1.** **Changes in elevation across months**. The dashed line indicates the chosen threshold of 1200 m a.s.l., indicating altitudinal movements.

**Figure S2.** **Activity range of Alpine choughs during behavioural observations in the town of Berchtesgaden.** Coloured polygons represent the 50% (red) and 95% (orange) minimum convex polygons (MCPs) derived from the birds' observed spatial locations. Water bodies are shown in blue, while anthropogenic features, including buildings and roads, are shown in black.

**Figure S3.** **Activity range estimates for 31 individuals**. Coloured lines represent the 50% Utilization Distribution (UD) for different individuals. Water bodies are shown in blue, anthropogenic structures such as buildings and roads are shown in black. The different shades of grey illustrate changes in elevation. For visualization purposes one individual is not shown.

**Figure S4**. **Map showing frequently revisited sites**. Polygons show the 100% MCPs inferred from the 50% top revisited sites for each individual (different colours for each individual). Water bodies are shown in blue, anthropogenic structures such as buildings and roads are shown in black. The different shades of grey illustrate changes in elevation.

**Figure S5.** **Visitation order across the four main human resources**. Shown are the visitation order across one month for four individuals (A-C). Colour represents the four different human resources (A=red, B=green, C=blue, D=lilac).

**Table S1.** Frequency of site revisitations by Alpine choughs, showing the number of times each location (A–D) was visited in first, second, third, and fourth order across revisitation sequences.

|  | **Order** | | | |
| --- | --- | --- | --- | --- |
| **Location** | **1** | **2** | **3** | **4** |
| **A** | 463 (90%) | 6 (2%) | 0 | 0 |
| **B** | 33 (6%) | 326 (79%) | 2 (1%) | 0 |
| **C** | 13 (3%) | 30 (7%) | 89 (63%) | 20 (95%) |
| **D** | 8 (1%) | 50 (12%) | 50 (36%) | 1 (5%) |
